# Hexanucleotide Repeat Expansion in *C9ORF72* Is Not Detected in the Treatment-Resistant Schizophrenia Patients of Chinese Han

**DOI:** 10.1371/journal.pone.0145347

**Published:** 2015-12-21

**Authors:** Xijia Xu, Shiping Xie, Xiaomeng Shi, Jie Lv, Xiaowei Tang, Xiaolan Wang, Shuiping Lu, Mingzhong Wang, Xiaobing Zhang, Jing Sun, Hui Yao

**Affiliations:** 1 Department of Psychiatry, Affiliated Nanjing Brain Hospital of Nanjing Medical University, Nanjing, 210029, China; 2 Nanjing Brain Hospital, Nanjing University Medical School, Nanjing, 210093, China; 3 Department of Psychiatry, Nanjing Qinglongshan Mental Hospital, Nanjing, 211123, China; 4 Department of Psychiatry, Affiliated Wutaishan Hospital of Medical College of Yangzhou University, Yangzhou, 225003, China; the Second Xiangya Hospital of Central South University, CHINA

## Abstract

Hexanucleotide (GGGGCC) repeat expansion in *C9ORF72* (HRE) causes frontotemporal lobar degeneration, frontotemporal dementia–amyotrophic lateral sclerosis, and amyotrophic lateral sclerosis. HRE was also seen in the genomes of patients suffering from several other degenerative diseases. However, whether it is present in the treatment-resistant schizophrenia patients remains unknown. Genotyping 386 patients suffering from treatment-resistant schizophrenia using the method of Repeat-Primed PCR, we reported here that no HRE was detected in the patients of Chinese Han.

## Introduction

Hexanucleotide (GGGGCC) repeat occurs in the first intron of human *C9ORF72* with different number of repeats usually fewer than 20. However, the hexanucleotide repeat expansion (HRE, greater than 30 repeats) causes FTLD (frontotemporal lobar degeneration), FTD−ALS (frontotemporal dementia–amyotrophic lateral sclerosis), and ALS [1–2] likely due to toxicity of the transcribed repeat, toxicity of protein dipeptides translated from the transcribed repeat, or loss of function of *C9ORF72* [1, 3–5]. The frequency of HRE is found to be varied in the patents from different populations. For example, it is present in 46.0% of familial ALS (fALS) and 21.1% of sporadic ALS (sALS) in the Finnish population [2], approximately 6% of sporadic and 25% of familial Caucasian FTLD cases [6], 27.1% of fALS and 3.2% of sALS in Spanish [7], only 0.3% of sALS [8] and no patients with fALS in Chinese [9], and 0.4% of sALS and no patients with fALS in Japanese [10]

Other than the neurodegenerative disorders like ALS and FTLD, HRE is also seen in the genomes of patients suffering from several other degenerative diseases such as Alzheimer disease [11–12] sporadic Creutzfeldt-Jakob disease, Huntington disease-like syndrome in the UK and Greece population [11–13], multiple system atrophy [14], rapid eye movement sleep behavior disorder [15], and depressive pseudodementia [16] though in rare cases.

Schizophrenia is an often devastating neuropsychiatric disorder that affects around 0.5–1% of the population [17]. Among the patients, about 30% of them are treatment resistant schizophrenia (TRS) that fail to respond adequately to the usual antipsychotic medications [17]. Schizophrenia is highly heritable but little is known about its pathophysiology. Recently, a research on a large population of patients with FTLD revealed that the presentation with late onset psychosis was significantly more frequent in HRE patients than in non-HRE ones [18] and 2 in 298 (0.67%) patients from Europe with schizophrenia or schizoaffective disorder were found to carry HRE [19]. However, whether HRE is present in TRS patients of Chinese Han or not remains unknown. To explore whether TRS patients of Chinese Han carry HRE, we detected the number of hexanucleotide (GGGGCC) repeat in 394 TRS patients and 337 healthy controls of Chinese Han.

## Materials and Methods

The researches were approved by Medical Ethics Committee of Nanjing Brain Hospital [No. (2011) LunShen (KY44)], and all clinical investigations were conducted according to the principles expressed in the Declaration of Helsinki. All control subjects participating in this study were adults and signed a written informed consent. The guardians of the patients suffering from TRS signed the written informed consent on behalf of the patients participating in this study according to Chinese law. The TRS patients (284 males and 110 females; age = 44.6 ± 10.7 years-old; age ranging from 16 to 71) were recruited from Affiliated Nanjing Brain Hospital of Nanjing Medical University, Nanjing Qinglongshan Mental Hospital, and Affiliated Wutaishan Hospital of Medical College of Yangzhou University in Jiangsu Province, China. The healthy controls (200 males and 137 females; age = 34.3 ± 10.2 years-old; age ranging from 20 to 89) were recruited from the Nanjing residents who participated in physical examination at Affiliated Nanjing Brain Hospital of Nanjing Medical University. The time when participants were recruited to the study was from November 2012 to September 2013. The diagnosis of TRS was made conforming to the criteria for TRS according to International Psychopharmacology Algorithm Project (IPAP, http://www.ipap.org/) by at least two psychiatry doctors who extensively interviewed the patients and reviewed their medical records. The patients had been documented poor functioning for 5 years at least, including lacking response to therapeutic trials of at least two antipsychotic drugs from two different chemical classes (or the medications had been administered for at least 4–6 weeks each at doses ≥ 400 mg equivalents of chlorpromazine or 5 mg/day of risperidone), and having moderate to severe psychopathology, especially positive symptoms, such as conceptual disorganization, suspiciousness, delusions, or hallucinatory behavior. The average duration of the illness of the patients was 22.2 ± 10.0 (n = 394) years.

To examine the number of the hexanucleotide repeat, we first isolated the genomic DNA from whole blood leukocytes collected from the subjects using a commercial kit (Qiagen, German). The hexanucleotide repeat in *C9ORF72* was PCR amplified in all patient and control cohorts using genotyping primers [1] with the forward primer (fluorescently labeled) of FAM-ACAGTACTCGCTGAGGGTGAAC and the reverse primer of GCGCAGGCACCGCAACCGCAG. The PCR reaction was carried out in a 10 μl mixture containing 2 μl of 5 × KAPA2G GC Buffer, 0.5 U of KAPA2G Robust HotStart DNA Polymerase, 0.4 μl of 10 mM dNTP, 0.15 μl of 10 μM forward primer and reverse primer and 1 μl of genomic DNA (50ng). The PCR conditions were 95°C 5 min, 27 cycles of (94°C 30 sec, 61°C 30 sec and 72°C 45 sec) and 60°C 30 min. The PCR was performed using GeneAmp 9600 (Applied Biosystems, USA). The PCR products were subjected to fragment length analysis on an automated 3730XL DNA-analyzer and the data were subjected to analysis using GeneMapperID v3.2 software (Applied Biosystems, USA).

To determine whether the single allele amplification of the hexanucleotide repeat with the genotyping primers was due to the presence of an un-amplifiable repeat expansion in the second allele of a subject, we used a repeat-primed PCR method specifically designed to the observed the hexanucleotide repeat as previously reported [1] with forward primer (fluorescently labeled) of FAM-ACAGTACTCGCTGAGGGTGAAC, reverse primer-MRX-R1 of CAGGAAACAGCTATGACCGGGCCCGCCCCGACCACGCCCCGGCCCCGGCCCCGG and repeats primer-M13R of CAGGAAACAGCTATGACC. The PCR reaction was carried out in a 10 μl mixture containing 1 μl of 10 × PCR Buffer, 0.2 μl of Roche FastStart Taq DNA polymerase, 2 μl of 5 × Q-solution, 0.2 μl of 10 mM dNTP, 0.18 mM 7-deaza-dGTP, 0.7 μl of dimethyl sulfoxide, 0.6 μl of 10 μM forward primer, 0.1 μl of 10 μM reverse primer-MRX-R1, 0.6 μl of 10 μM reverse primer-M13R and 1μl of genomic DNA (50ng). The PCR was performed in GeneAmp 9600 (Applied Biosystems, USA) using a touchdown cycling program. The PCR conditions were 95°C for 5 min, 15 cycles of (95°C 1 min, the annealing temperature gradually lowered from 70°C to 56°C in 1°C decrement with a 3 min extension time at 72°C for each cycle), 25 cycles of (95°C 1 min, 56°C 1 min and 72°C 3 min), followed by 72°C extension for 60 min. The PCR products were subjected to analysis as described above. HRE was identified by a characteristic saw tooth pattern with a 6 bp periodicity [2].

## Results and Discussions

**Fig 1:**
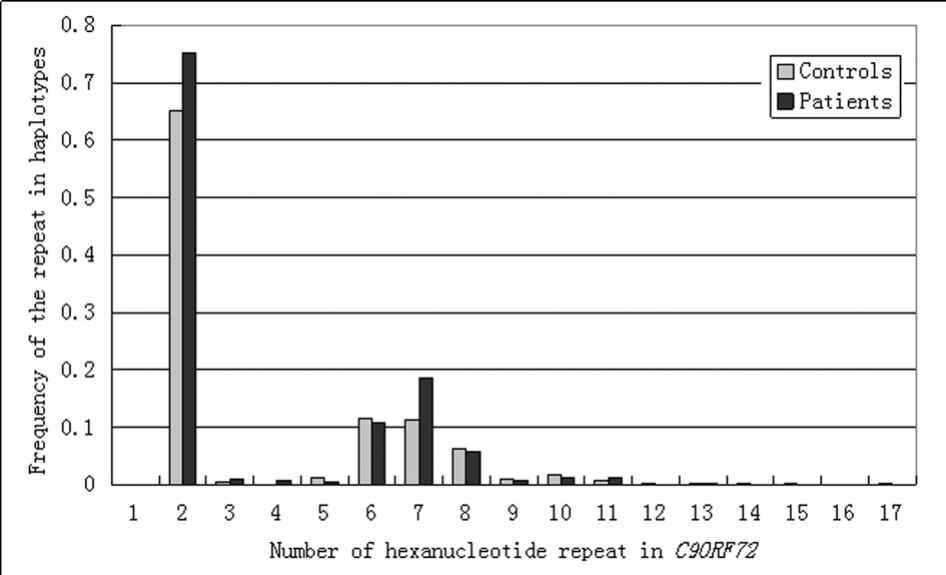
Distribution of different number of hexanuclotide repeats in *C9ORF792* of treatment-resistant schizophrenia patients and controls of Chinese Han. 772 haplotypes from 386 treatment-resistant schizophrenia patients and 664 haplotypes from 332 control cohorts were genotyped for their carrying the number of hexanuclotide repeat in *C9ORF792*. The X-axis shows the number of hexanuclotide repeat in *C9ORF792* and the Y-axis denotes the frequency of the repeats in the haplotypes. The frequency was calculated as the number of haplotypes carrying the hexanucleotide repeats divided by the number of total haplotypes.

Totally, we obtained 2904 hexanucleotide repeats from 386 TRS patients and 2531 ones from 332 control cohorts. The genotyping success rate was 97.97% of TRS patients and 98.52% of controls. We did not find any abnormal HRE in TRS patients of Chinese Han (Fig 1).

The largest number of hexanucleotide repeats was 17. It was found in allele 2 of control cohorts. The number of the hexanucleotide repeats (Average ± STDEV) in TRS patients was 2.52 ± 1.45 (allele 1) and 5.00 ± 2.77 (allele 2) whereas the one in control cohorts was 2.57 ± 1.51 (allele 1) and 5.13 ± 3.15 (allele 2), respectively. Performing Student *t*-test analysis on the number of the hexanucleotide repeats, we found there was no significant difference at allele 1 and allele 2 between TRS patients and control cohorts of Chinese Han, respectively. The results are consistent with the recent report revealing that no abnormal HRE was detected (0 of 466) in Japanese schizophrenia patients [20]. Current literature demonstrates that the mutation of HRE originated in the Finnish population and then spread all over Europe [21]. This founder effect could be the reason for its absence in non-Caucasian population [20]. Consistent with the hypothesis, HRE was highly found in European population [1–2, 6–7] whereas it was seldom found in Asian population [9–10].

## References

[1] DeJesus-Hernandez M, Mackenzie IR, Boeve BF, Boxer AL, Baker M, Rutherford NJ, et al Expanded GGGGCC hexanucleotide repeat in noncoding region of C9ORF72 causes chromosome 9p-linked FTD and ALS. Neuron. 2011 10 20;72(2):245–56. 10.1016/j.neuron.2011.09.011 21944778 PMC3202986

[2] Renton AE, Majounie E, Waite A, Simo´n-Sa´nchez J, Rollinson S, Gibbs JR, et al A hexanucleotide repeat expansion in C9ORF72 is the cause of chromosome 9p21-linked ALS-FTD. Neuron. 2011 10 20;72(2):257–68. 10.1016/j.neuron.2011.09.010 21944779 PMC3200438

[3] Donnelly CJ, Zhang PW, Pham JT, Heusler AR, Mistry NA, Vidensky S, et al RNA toxicity from the ALS/FTD C9ORF72 expansion is mitigated by antisense intervention. Neuron. 2013 10 16;80(2):415–28. 10.1016/j.neuron.2013.10.015 24139042 PMC4098943

[4] Gijselinck I, Van Langenhove T, van der Zee J, Sleegers K, Philtjens S, Kleinberger G, et al A C9orf72 promoter repeat expansion in a Flanders-Belgian cohort with disorders of the frontotemporal lobar degeneration-amyotrophic lateral sclerosis spectrum: a gene identification study. Lancet Neurol. 2012 1;11(1):54–65. 10.1016/S1474-4422(11)70261-7 22154785

[5] Mori K, Weng SM, Arzberger T, May S, Rentzsch K, Kremmer E, et al The C9orf72 GGGGCC repeat is translated into aggregating dipeptide-repeat proteins in FTLD/ALS. Science. 2013 3 15;339(6125):1335–8. 10.1126/science.1232927 23393093

[6] Rademakers R. C9orf72 repeat expansions in patients with ALS and FTD. Lancet Neurol. 2012 4;11(4):297–8. 10.1016/S1474-4422(12)70046-7 22406229 PMC4114244

[7] García-Redondo A, Dols-Icardo O, Rojas-García R, Esteban-Pérez J, Cordero-Vázquez P, Muñoz-Blanco JL, et al Analysis of the C9orf72 gene in patients with amyotrophic lateral sclerosis in Spain and different populations worldwide. Hum Mutat. 2013 1;34(1):79–82. 10.1002/humu.22211 22936364

[8] He J, Tang L, Benyamin B, Shah S, Hemani G, Liu R, et al C9orf72 hexanucleotide repeat expansions in Chinese sporadic amyotrophic lateral sclerosis. Neurobiol Aging. 2015 9;36(9):2660e1-8. 10.1016/j.neurobiolaging.2015.06.002 26142124

[9] Liu R, Tang L, Cai B, Liu X, Ye S, Ma Y, et al C9orf72 repeat expansions are not detected in Chinese patients with familial ALS. Amyotroph Lateral Scler Frontotemporal Degener. 2013 12;14(7–8):630–1. 10.3109/21678421.2013.817588 23869403

[10] Ogaki K, Li Y, Atsuta N, Tomiyama H, Funayama M, Watanabe H, et al Analysis of C9orf72 repeat expansion in 563 Japanese patients with amyotrophic lateral sclerosis. Neurobiol Aging. 2012 10;33(10):2527e11-6. 10.1016/j.neurobiolaging.2012.05.011 22727276

[11] Beck J, Poulter M, Hensman D, Rohrer JD, Mahoney CJ, Adamson G, et al Large C9orf72 hexanucleotide repeat expansions are seen in multiple neurodegenerative syndromes and are more frequent than expected in the UK population. Am J Hum Genet. 2013 3 7;92(3):345–53. 10.1016/j.ajhg.2013.01.011 23434116 PMC3591848

[12] Kohli MA, John-Williams K, Rajbhandary R, Naj A, Whitehead P, Hamilton K, et al Repeat expansions in the C9ORF72 gene contribute to Alzheimer's disease in Caucasians. Neurobiol Aging. 2013 5;34(5):1519e5-12. 10.1016/j.neurobiolaging.2012.10.003 PMC3586789 23107433

[13] Koutsis G, Karadima G, Kartanou C, Kladi A, Panas M. C9ORF72 hexanucleotide repeat expansions are a frequent cause of Huntington disease phenocopies in the Greek population. Neurobiol Aging. 2015 1;36(1):547e13-6. 10.1016/j.neurobiolaging.2014.08.020 25248608

[14] Goldman JS, Quinzii C, Dunning-Broadbent J, Waters C, Mitsumoto H, Brannagan TH 3rd, et al Multiple system atrophy and amyotrophic lateral sclerosis in a family with hexanucleotide repeat expansions in C9orf72. JAMA Neurol. 2014 6;71(6):771–4. 10.1001/jamaneurol.2013.5762 24733620 PMC4051831

[15] Daoud H, Postuma RB, Bourassa CV, Rochefort D, Gauthier MT, Montplaisir J, et al C9orf72 repeat expansions in rapid eye movement sleep behaviour disorder. Can J Neurol Sci. 2014 11;41(6):759–62. 10.1017/cjn.2014.39 25377888

[16] Bieniek KF, van Blitterswijk M, Baker MC, Petrucelli L, Rademakers R, Dickson DW. Expanded C9ORF72 hexanucleotide repeat in depressive pseudodementia. JAMA Neurol. 2014 6;71(6):775–81. 10.1001/jamaneurol.2013.6368 24756204 PMC4197801

[17] Frank J, Lang M, Witt SH, Strohmaier J, Rujescu D, Cichon S, et al Identiication of increased genetic risk scores for schizophrenia in treatment-resistant patients. Mol Psychiatry. 2015 2;20(2):150–1. 10.1038/mp.2014.56 24888364 PMC4356742

[18] Galimberti D, Fenoglio C, Serpente M, Villa C, Bonsi R, Arighi A, et al Autosomal dominant frontotemporal lobar degeneration due to the C9ORF72 hexanucleotide repeat expansion: late-onset psychotic clinical presentation. Biol Psychiatry. 2013 9 1;74(5):384–91. 10.1016/j.biopsych.2013.01.031 Epub 2013 Mar 7. 23473366

[19] Galimberti D, Reif A, Dell'osso B, Kittel-Schneider S, Leonhard C, Herr A, et al C9ORF72 hexanucleotide repeat expansion is a rare cause of schizophrenia. Neurobiol Aging. 2014 5;35(5):1214e7-1214.e10. 10.1016/j.neurobiolaging.2013.12.004 24387986

[20] Yoshino Y, Mori Y, Ochi S, Numata S, Ishimaru T, Yamazaki K, et al No abnormal hexanucleotide repeat expansion of C9ORF72 in Japanese schizophrenia patients. J Neural Transm (Vienna). 2015 5;122(5):731–2. 10.1007/s00702-014-1295-y 25115936

[21] Pliner HA, Mann DM, Traynor BJ. Searching for Grendel: origin and global spread of the C9ORF72 repeat expansion. Acta Neuropathol. 2014 3;127(3):391–6. 10.1007/s00401-014-1250-x 24496499 PMC4545603

